# Supplementary material for: Resolving the full spectrum of human genome variation using Linked-Reads
Source: Genome Res. 2019 Apr;29(4):635–45. doi: 10.1101/gr.234443.118 (PMC6442396; doi:10.1101/gr.234443.118)
Supplement: Supplemental Material [file supp_gr.234443.118_Supplemental_longranger-2.2.zip › longranger-2.2.tar.bz2!/lr2.2-public/lib/stan/cmdstan/stan/doc/api/html/matrix__error__handling_8hpp_source.html]

Stan: src/stan/math/matrix\_error\_handling.hpp Source File


|  |  |
| --- | --- |
|  | Stan  2.5.0  probability, sampling & optimization |


- Main Page
- Related Pages
- Namespaces
- Classes
- Files

- File List
- File Members

All Classes Namespaces Files Functions Variables Typedefs Enumerations Enumerator Friends Macros Pages

- src
- stan
- math

matrix\_error\_handling.hpp

Go to the documentation of this file.

1 #ifndef STAN\_\_MATH\_\_MATRIX\_ERROR\_HANDLING\_HPP

2 #define STAN\_\_MATH\_\_MATRIX\_ERROR\_HANDLING\_HPP

3

4 #include <stan/math/error\_handling/matrix/check\_cholesky\_factor.hpp>

5 #include <stan/math/error\_handling/matrix/check\_cholesky\_factor\_corr.hpp>

6 #include <stan/math/error\_handling/matrix/check\_column\_index.hpp>

7 #include <stan/math/error\_handling/matrix/check\_corr\_matrix.hpp>

8 #include <stan/math/error\_handling/matrix/check\_cov\_matrix.hpp>

9 #include <stan/math/error\_handling/matrix/check\_ldlt\_factor.hpp>

10 #include <stan/math/error\_handling/matrix/check\_lower\_triangular.hpp>

11 #include <stan/math/error\_handling/matrix/check\_matching\_dims.hpp>

12 #include <stan/math/error\_handling/matrix/check\_matching\_sizes.hpp>

13 #include <stan/math/error\_handling/matrix/check\_multiplicable.hpp>

14 #include <stan/math/error\_handling/matrix/check\_nonzero\_size.hpp>

15 #include <stan/math/error\_handling/matrix/check\_ordered.hpp>

16 #include <stan/math/error\_handling/matrix/check\_pos\_definite.hpp>

17 #include <stan/math/error\_handling/matrix/check\_pos\_semidefinite.hpp>

18 #include <stan/math/error\_handling/matrix/check\_positive\_ordered.hpp>

19 #include <stan/math/error\_handling/matrix/check\_range.hpp>

20 #include <stan/math/error\_handling/matrix/check\_row\_index.hpp>

21 #include <stan/math/error\_handling/matrix/check\_simplex.hpp>

22 #include <stan/math/error\_handling/matrix/check\_size\_match.hpp>

23 #include <stan/math/error\_handling/matrix/check\_spsd\_matrix.hpp>

24 #include <stan/math/error\_handling/matrix/check\_square.hpp>

25 #include <stan/math/error\_handling/matrix/check\_std\_vector\_index.hpp>

26 #include <stan/math/error\_handling/matrix/check\_symmetric.hpp>

27 #include <stan/math/error\_handling/matrix/check\_unit\_vector.hpp>

28 #include <stan/math/error\_handling/matrix/check\_vector.hpp>

29 #include <stan/math/error\_handling/matrix/constraint\_tolerance.hpp>

30 #include <stan/math/error\_handling/matrix/validate\_non\_negative\_index.hpp>

31

32 #endif

check\_pos\_definite.hpp

check\_simplex.hpp

check\_matching\_dims.hpp

check\_multiplicable.hpp

check\_positive\_ordered.hpp

check\_size\_match.hpp

check\_column\_index.hpp

constraint\_tolerance.hpp

check\_cholesky\_factor\_corr.hpp

check\_pos\_semidefinite.hpp

check\_matching\_sizes.hpp

check\_lower\_triangular.hpp

check\_std\_vector\_index.hpp

check\_symmetric.hpp

check\_cov\_matrix.hpp

validate\_non\_negative\_index.hpp

check\_row\_index.hpp

check\_ldlt\_factor.hpp

check\_ordered.hpp

check\_unit\_vector.hpp

check\_corr\_matrix.hpp

check\_cholesky\_factor.hpp

check\_vector.hpp

check\_nonzero\_size.hpp

check\_range.hpp

check\_spsd\_matrix.hpp

check\_square.hpp


---

[ Stan Home Page ]

*© 2011–2014,
Stan Development Team.*
